# Use of the Hypertension Self-Care Profile: A Scoping Review

**DOI:** 10.3390/ijerph22081244

**Published:** 2025-08-08

**Authors:** Hae-Ra Han, Chitchanok Benjasirisan, Faith E. Metlock, Yordanos Tesfai, Yvonne Commodore-Mensah

**Affiliations:** 1 School of Nursing, Johns Hopkins University, Baltimore, MD 21205, USA; 2 Bloomberg School of Public Health, Johns Hopkins University, Baltimore, MD 21205, USA

**Keywords:** self-care, hypertension self-care profile, behaviors, self-efficacy, motivation, scoping review, validity, reliability, outcome measure

## Abstract

Self-care is a critical component of chronic disease management and is linked to better health outcomes. The Hypertension Self-Care Profile (HBP SCP) is one of the few validated instruments designed to assess not only behaviors but also self-efficacy and motivation in HBP self-care. This scoping review synthesized published research using the HBP SCP to examine its scope and utility across diverse populations. A total of 48 studies were reviewed—34 non-validation studies and 14 validation studies—spanning regions including Asia, the Middle East, and the Americas. The HBP SCP showed strong psychometric performance across multiple cultural adaptations, with Cronbach’s alpha values ranging from 0.73 to 0.99. Several correlates of HBP self-care emerged, including self-efficacy, social support, health literacy, and education. Findings also revealed that HBP self-care remains suboptimal, particularly among rural populations and low- and middle-income countries. The HBP SCP has proven to be a versatile and culturally adaptable instrument for evaluating HBP self-care behaviors, self-efficacy, and motivation. Its consistently demonstrated validity and reliability across diverse contexts, combined with its responsiveness in randomized controlled trials, affirm its value as both a clinical assessment tool and a research outcome measure in interventions aimed at improving cardiovascular health.

## 1. Introduction

High blood pressure (HBP) is a major risk factor that can cause serious cardiovascular health consequences such as stroke, coronary heart disease, heart failure, and kidney disease [1]. HBP is also associated with other debilitating outcomes including dementia [2]. Globally, an estimated 1.28 billion adults aged 30–79 years have HBP, defined as systolic blood pressure (BP) ≥ 140 mm Hg and/or diastolic BP ≥ 90 mm Hg [3]. According to a report using data from the National Health and Nutrition Examination Survey (NHANES) 2017–2020, nearly half of adults (48.1% or 119.9 million) in the United States (US) have HBP [4].

Despite effective pharmacological and nonpharmacological treatment, BP control (systolic BP < 140 mm Hg and diastolic BP < 90 mm Hg) remains suboptimal; per recent research, even declining. For example, Muntner et al. [5] analyzed data from NHANES 2009 to 2012, 2013 to 2016, and 2017 to 2020 and found that 52.8%, 51.3%, and 48.2% of US adults with HBP had controlled BP, in the respective periods. Data from NHANES 2017–2020 revealed that compared with non-Hispanic White adults, BP control was less likely among non-Hispanic Black, non-Hispanic Asian, and Hispanic adults (25.1% versus 16.8%, 18.4%, and 17.2%, respectively) [4].

Self-care is an integral part of chronic disease management and has been linked to better health outcomes, including BP control [6,7,8,9]. Within the context of HBP, self-care areas encompass non-smoking, weight management, exercise, low-sodium and low-fat diet, decreased alcohol consumption, monitoring of BP, stress reduction, and other adherence activities such as regular physician visits and medication taking [10,11]. As such, existing tools addressing HBP self-care evaluate some (e.g., Hill–Bone Adherence Scale, Morisky Medication Scale, or Hypertension Self-Care Activity Level Effects) [12,13] or all of these areas (e.g., Hypertension Self-Care Profile [HBP SCP]) [12]. In particular, guided by two theoretical approaches—Orem’s Self-Care model and Motivational Interviewing—HBP SCP is one of the few validated instruments designed to measure not only behavior but also self-efficacy and motivation in terms of HBP self-care, hence allowing researchers to use one or more of the relevant scales per their study focus.

Since the original validation study was published in 2014 [11], HBP SCP has been used in multiple studies with various designs and samples. A few reviews were done to include HBP SCP and other instruments addressing self-efficacy in cardiovascular disease or self-management for adults with diverse chronic conditions such as diabetes, HIV/AIDS, heart disease/HBP and arthritis [14,15,16]. None of the published reviews specifically looked at HBP SCP, nor did they comprehensively examine the psychometrics of the instrument across the empirical studies conducted. Given the ongoing popularity and continuing use of HBP SCP, the purpose of this review was to identify published research using HBP SCP and synthesize the findings from the studies to better understand the scope and utility of HBP SCP as an instrument measuring HBP self-care behavior, motivation and self-efficacy across diverse settings, populations, and languages.

## 2. Materials and Methods

### 2.1. Design and Search

We conducted a scoping review [16] to identify, select, and synthesize relevant studies using the HBP SCP. In consultation with a medical librarian, the search strategy was defined by aggregating various search terms that describe the HBP SCP instrument. The terms “High Blood Pressure Self-Care Profile”, “Hypertension Self-Care Profile”, “Self-Care Profile”, and “Hypertension Self-Care” were entered into the following databases: PubMed, Scopus, Google Scholar, Embase, CINAHL, Web of Science, and Cochrane. We conducted an initial search on 30 October 2023, and another search on 11 March 2024, to cover more recent publications, which resulted in the final search to include articles published from January 2014 to March 2024. A manual search was also performed in March 2024 to identify any additional relevant articles. This review was conducted in accordance with the Preferred Reporting Items for Systematic Reviews and Meta-Analyses (PRISMA) guidelines.

### 2.2. Selection of Studies

**Figure 1:**
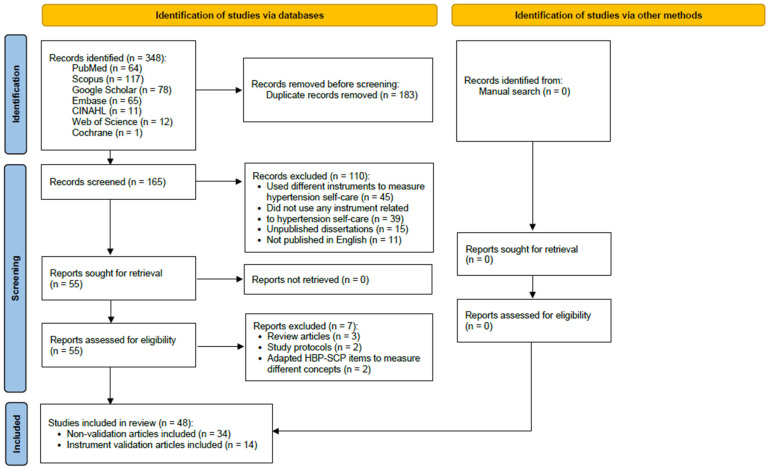
PRISMA 2020 flow diagram for new systematic reviews which included searches of databases and other sources [21]. This work is licensed under CC BY 4.0. To view a copy of this license, visit https://creativecommons.org/licenses/by/4.0/ (accessed on 5 August 2025).

Figure 1 illustrates the flow of our study selection process. We included peer-reviewed studies published in English that utilized one or more of the HBP SCP scales. Specifically, studies that validated psychometric properties, provided translation of the instrument, or utilized the HBP SCP as a study variable or outcome measurement were included. Additionally, non-English papers were included if an accompanying abstract was written in English and provided sufficient data for extraction. Excluded literature included review articles, study protocols, and studies that did not utilize the instrument. The initial search yielded 173 records, with 175 additional records from a second search, totaling 348. After removing duplicates, 165 titles and abstracts were screened for relevance. Each record was independently reviewed by two of the three authors (CB, FEM, and YT). Discrepancies were resolved by consensus among the authors. A total of 110 records were deemed ineligible for the following reasons: use of other instruments to measure HBP self-care (*n* = 45), did not use any instrument related to HBP self-care (*n* = 39), unpublished dissertations (*n* = 15), and non-English publications (*n* = 11). The remaining 55 records underwent full-text review. Of the 55 full-text articles, three were review papers [14,15,16], two were study protocols without data [17,18], and two studies used some of the items on the HBP SCP to create a new instrument measuring different concepts such as dietary regulation [19] or knowledge [20]. Ultimately, 48 studies were included in the analysis.

### 2.3. Data Extraction

Preliminary data extraction was conducted and included study-specific information including author and publication year, country of origin, language, study design, study sample demographics, number and method of instrument administration, psychometrics properties and main findings. All the initial extraction and discrepancies were rectified by the first senior author (HRH). Subsequently, two authors (CB and FEM) synthesized and organized data based on whether instrument validation was a primary goal of the literature and presented this information in the tables.

## 3. Results

### 3.1. Characteristics of Studies

This review includes 48 studies that used the HBP SCP. All non-validation studies (*n* = 34) are summarized in Table 1 and the instrument validation studies (*n* = 14) in Table 2. The majority of the non-validation studies used descriptive or correlational study design (*n* = 18) [22,23,24,25,26,27,28,29,30,31,32,33,34,35,36,37,38,39] followed by clinical trials using either quasi-experimental (*n* = 8) [40,41,42,43,44,45,46,47] or experimental study designs (*n* = 6) [48,49,50,51,52,53]. One study [54] used case-control design, and one study [55] examined the psychometric properties of the Chinese version of the Dyadic Relationship Scale, using the HBP SCP to provide evidence for convergent validity. The validation studies included 14 studies that translated, adapted, or validated the HBP SCP for various cultural and linguistic contexts [56,57,58,59,60,61,62,63,64,65,66,67,68,69].

The studies represented a diverse geographical spread: twenty-two studies were conducted in Southeast Asia (e.g., the Philippines, Vietnam, Indonesia, Singapore, Malaysia, and Thailand) [25,29,33,34,35,36,37,38,39,43,46,47,49,50,54,55,60,63,64,65,66,67], ten in the Middle East (Saudi Arabia, Iran, and Turkey) [23,24,26,44,51,56,57,58,59,68], eight in the Americas (the US and Brazil) [28,31,32,40,41,45,48,52], six in East Asia (the Republic of Korea and China) [30,42,53,61,62,69], and two in South Asia (Nepal and Bhutan) [22,27]. A total of 10,251 participants were included in these studies with sample sizes ranging from 11 to 1123. The majority of the participant samples consisted of adult patients with HBP (*n* = 40), but also included healthy populations (*n* = 3) [25,30,37] and others with chronic conditions such as stroke (*n* = 2) [50,54], HBP with complications (*n* = 1) [35], chronic kidney disease (*n* = 1) [29], and peripheral neuropathy (*n* = 1) [31].

### 3.2. Use of HBP SCP Scales

Most studies used one of the three scales included in the HBP SCP—behaviors, motivation, and self-efficacy. Eight studies examined all three scales of the HBP SCP in primary care or community-based clinical settings across diverse countries [23,29,35,36,39,46,48,49]. Detailed results of the non-validation studies can be found in Table S1.

#### 3.2.1. Behavior Scale

The behavior scale was most frequently used, appearing in 23 of 34 non-validation studies [22,23,24,25,26,27,29,30,32,33,35,36,37,38,39,41,42,46,47,48,49,52,55]. Studies employed cross-sectional designs [22,23,24,25,26,27,29,30,32,33,35,36,37,38,39,46], quasi-experimental studies [41,42,47,52], and randomized controlled trials [48,49,53]. Most were conducted in primary care or community settings, including urban clinics [33,39,49,52] and rural health centers [25,53], with fewer targeting specialized populations [26,32,41,52].

AlHadlaq et al. [23] found less than half of Saudi patients engaged in BP self-monitoring (37.9%) or dietary modifications (35.8%), while adherence to smoking cessation (65.2%) and physician visits (85.0%) was higher. Dorji et al. [27] noted Bhutanese participants occasionally practiced lifestyle modifications, with smoking cessation scoring higher (mean = 3.3) than dietary or physical activity behaviors. Visanuyothin et al. [38] reported only 13% of Thai participants demonstrated adequate HBP self-care behaviors. Buco et al. [25] observed HBP self-care was significantly associated with quality of life in rural Philippines (β = 0.15, *p* = 0.016).

Studies identified factors influencing HBP self-care behaviors. Bahari et al. [56] found family support and self-efficacy positively associated with self-care behaviors among Saudi men (β = 0.63, *p* < 0.05). Ho et al. [29] reported health literacy significantly linked to behavior scores among Singaporean adults with HBP and chronic kidney disease (β = 0.30, *p* < 0.001). Lunyera et al. [32] found improved perceived neighborhood health significantly impacted behavior scores among African Americans in Baltimore. Higher education and family support consistently predicted better self-care behaviors. Kim et al. [31] found significant correlations between higher education and improved HBP self-care in US online participants. Salim et al. [36] revealed higher education levels correlated with better adherence in Malaysian primary care patients, with education positively associated with HBP SCP scores (*p* < 0.001).

Interventions to improve HBP self-care behaviors showed promising results. Moon et al. [49] compared smartphone app intervention with traditional logbooks in Singapore, finding no significant differences in behavior scores (*p* = 0.43) but higher adherence to BP monitoring among older app users. Zabler et al. [52] observed significant improvements in behavior scores over six months among low-income African American patients, with scores increasing from baseline (mean = 46.73) to follow-up (mean = 54.27, *p* < 0.001). Zeng et al. [53] demonstrated significant post-intervention improvements in rural Chinese patients (F = 17.668, *p* < 0.001). Visanuyothin et al. [47] showed that a six-month health literacy intervention improved behavior scores in Thai urban participants (mean = 2.4 to 2.7, *p* < 0.001).

#### 3.2.2. Self-Efficacy Scale

The self-efficacy scale appeared in 19 of 34 non-validation studies [23,28,29,32,35,36,39,40,41,43,44,45,46,48,49,50,53,54,55] across primary care clinics [23,29,39], community clinics [45,48], and rural settings [40]. Self-efficacy consistently influenced HBP self-care behaviors. Ho et al. [29] and Wee et al. [39] found higher self-efficacy scores associated with improved self-care behaviors, particularly medication adherence and lifestyle modifications. Zeng et al. [53] demonstrated that self-efficacy improvements significantly enhanced adherence to diet, medication, and exercise in rural Chinese populations. Boulware et al. [48] reported that intervention participants achieved significantly higher self-efficacy and behavior scores than usual care. Lunyera et al. [32] observed better self-efficacy linked to improved medication adherence and BP monitoring among African Americans. Gardiner et al. [40] found positive relationships between self-efficacy and dietary/physical activity adherence in older adults. Türkoğlu et al. [44] reported significant self-efficacy increases following educational interventions. Bahari et al. [56] and Pahria et al. [35] found that self-efficacy mediated relationships between social support and self-care behaviors. Han et al. [41] reported that culturally tailored interventions for Spanish-speaking Latinos improved self-efficacy significantly. Salim et al. [36] found men had lower self-efficacy scores than women. Rasyid et al. [54] reported that lower self-efficacy scores increased risk of poor HBP outcomes. Zeng et al. [55] found a strong negative correlation between the HBP SCP self-efficacy scale and Dyadic Relationship Scales (r = −0.70, *p* < 0.001).

#### 3.2.3. Motivation Scale

The motivation scale was used less frequently, appearing in 10 of 34 non-validation studies [23,29,35,36,39,46,48,49]. Most studies were conducted in primary care or community settings [23,29,36,39], with interventions in community clinics [48] and digital platforms [49]. AlHadlaq et al. [23] reported low engagement in physical activity (12.8%) but moderate motivation for healthier cooking (60.9%) and limiting high-salt condiments (56.7%). Ho et al. [29] observed significant positive association between motivation and self-care behaviors (β = 0.40, *p* < 0.001). Wee et al. [39] identified education and family support as factors contributing to higher motivation scores. Pahria et al. [35] demonstrated motivation as a significant predictor of adherence among Indonesian patients with hypertensive complications. Boulware et al. [48] observed that patients in a problem-solving intervention had significantly higher motivation scores, improving self-care behaviors versus usual care (OR = 18.7, 95% CI = 4.0–87.3). Moon et al. [49] found no significant differences in motivation between smartphone app and traditional logbook groups, though both experienced slight post-intervention increases.

### 3.3. Psychometrics of HBP SCP

Fourteen of the 48 studies included psychometric information. Most of the validation studies focused on translating and validating measurements (*n* = 12), while two studies involved validation using either web-based approaches [60] or comparison between the Self-Care of Hypertension Inventory and HBP SCP [69]. Most studies employed forward and backward translation methods to ensure the tool’s adaptability to local populations (*n* = 11). One study [61] utilized Cha’s combined translation technique [70], which includes forward–backward translation, bilingual techniques, group discussions, and pretest strategies. The full measurement has been translated into various languages, including Arabic [56], Persian [57], Turkish [59,68], Chinese [61], Indonesian [64,66], Vietnamese [67], and Singaporean contexts [60,63,65]. Some studies focused on translating and validating specific subscales. For example, the behavioral and self-efficacy scales were validated in Arabic [56], the self-efficacy scale in Persian [58], and the behavioral scale in Korean [62]. Psychometric properties from validation studies of HBP SCP are presented in Table S2.

#### 3.3.1. Validity

HBP SCP validity was tested among adult patients across community [66], public places [58], senior welfare centers [62], community health centers [57,61], primary care clinics [56,59,60,63,64,65], outpatient clinics [67,68], and in-hospital settings [61,69]. Approaches included content validity index, factor analyses, and concurrent/criterion validity testing.

Nine studies evaluated content validity through expert review panels, with content validity indexes ranging from 0.80 to 0.99. Upoyo et al. [66] achieved CVI of 0.96 for the Indonesian version. Versiyonunun et al. [68] reported mean CVI of 0.99 for behavior, 0.98 for motivation, and 0.96 for self-efficacy in the Turkish version. 

Construct validity was assessed in nine studies using exploratory or confirmatory factor analysis [56,58,59,61,62,64,67,68,69]. Bahari et al. [56] found Arabic version factor loadings for the behavior scale (0.26–0.70) and self-efficacy scale (0.29–0.77), though three behavior items and one self-efficacy item fell below the 0.30 threshold. The Persian version identified three factors with loadings ranging from dietary (0.47–0.74), disease management (0.36–0.68), and treatment adherence (0.56–0.63), demonstrating good model fit (NNFI = 0.90, CFI = 0.91, IFI = 0.91, RMSEA = 0.082) [58]. The Turkish version explained 46.32% variance for behavior, 45.87% for motivation, and 48.08% for self-efficacy scales, with factor loadings of 0.62–0.78, 0.54–0.80, and 0.57–0.77, respectively [59]. The Chinese version identified six behavior factors, with health promotion (63.15%) and habit modification (10.01%) as primary contributors [61]. The Vietnamese version showed standardized factor loadings from 0.35 to 0.96 with acceptable convergent validity [67].

Na et al. [62] tested the concurrent validity of the Korean behavior scale, finding significant correlations with self-efficacy (r = 0.32) and HBP treatment compliance (r = 0.39). Zhao et al. [69] demonstrated criterion validity by finding strong correlation (r = 0.70) between the Chinese HBP SCP and Exercise of Self-Care Agency Scale among 200 older Chinese adults.

#### 3.3.2. Reliability

Studies with psychometric information reported reliability using Cronbach’s alpha (*n* = 14) [56,57,58,59,60,61,62,63,64,65,66,67,68,69], Guttman split-half reliability (*n* = 2) [68,69], and Spearman–Brown reliability (n = 1) [68]. Cronbach’s alpha values were consistently high, ranging from 0.73 to 0.99 for total scale and 0.81–0.96 for behavior, 0.88–0.95 for *motivation*, and 0.78–0.95 for self-efficacy subscales. Some studies reported very high values (>0.95) for total scale [60,69], self-efficacy [60,65], and behavior subscales [66].

The Vietnamese version identified five behavior factors with overall Cronbach’s alpha of 0.79 and subscale values of 0.69 for advanced self-management skills, 0.82 for adverse health behaviors, 0.76 for medication adherence, 0.73 for diet-related knowledge, and 0.68 for information skills [67]. Split-half reliability demonstrated good internal consistency with coefficients of 0.83 for behavior, 0.94 for motivation, 0.75 for self-efficacy for the Turkish version [68], and 0.91 for total scale for the Chinese version [69]. Spearman–Brown reliability coefficients for the Turkish version were 0.85 for behavior, 0.96 for motivation, and 0.75 for self-efficacy [68].

Test–retest reliability was assessed in eight studies [57,58,59,60,61,63,65,67] with ICC values of 0.66–0.95 for behavior, 0.66–0.98 for motivation, 0.67–0.93 for self-efficacy, and 0.93 for total scale. The Malaysian version tested among Singaporean adults reported the lowest ICC values (0.66 for behavior, 0.66 for motivation, 0.68 for self-efficacy), likely due to low online response rate (43%) and lack of incentives [65]. Gheshlagh et al. [58] reported higher test–retest reliability with ICC of 0.93 for the total scale over two weeks among 260 patients in Tehran.

## 4. Discussion

This review synthesized current evidence on the use of the HBP SCP and its role in evaluating HBP self-care behaviors, self-efficacy, and motivation across diverse populations, settings, and study designs. Across studies, the HBP SCP demonstrated consistent associations with BP control and self-care adherence outcomes, highlighting its sensitivity to change over time and utility in intervention research. Additionally, we found that the HBP SCP has been widely adapted into various languages, with the translated versions showing strong psychometric properties.

The HBP SCP proved to be a versatile instrument, used successfully in cross-sectional, quasi-experimental, and RCT designs. In particular, RCTs leveraging the HBP SCP as a primary or secondary outcome measure demonstrated its responsiveness to interventions aimed at improving self-care. Studies showed significant pre- and post-intervention differences in HBP self-care behavior scores, indicating its effectiveness in detecting behavioral changes among low-income African Americans in the US [48,52], adults with HBP in rural China [53], patients with stroke [54], or housewives with HBP in Indonesia [43], community-residing adults with HBP in Malaysia [46], and individuals in urban communities in Thailand [47]. Both Boulware et al. [48] and Yatim et al. [46] also observed significant improvements in self-efficacy and motivation among the intervention participants. Similarly, Türkoğlu et al. [44] and Zeng et al. [53] found improved self-efficacy in managing HBP among adult patients with HBP in Turkey and rural China, respectively. Taken together, this highlights the value of the HBP SCP as both a clinical assessment tool and a research outcome measure in intervention trials aiming to reduce cardiovascular risk through improved HBP self-management. Future research is warranted to investigate how the HBP SCP compares to other established tools in its ability to detect differences in HBP self-care across patient subgroups and to identify individuals at risk for poor BP control due to inadequate self-care. Such investigations could help determine its relative utility and inform its broader adoption in practice.

The findings revealed that overall levels of HBP self-care remain suboptimal, particularly among rural populations and in studies conducted in low- and middle-income countries [22,27,37,38]. These studies reported low scores in key self-care areas such as medication adherence, physical activity, and dietary modifications, highlighting persistent challenges in translating knowledge into behavior. Barriers identified in these settings included limited access to healthcare resources—including medication—low health literacy, poor patient–provider communication, and a shortage of skilled providers [71,72,73,74]. These challenges underscore the need for more context-specific interventions and support systems to improve HBP self-care. Promising strategies include expanding non-communicable disease services through community pharmacies, implementing community outreach programs using mobile health interventions and community health workers, and mobilizing telehealth systems—approaches that have demonstrated effectiveness in enhancing HBP management in under-resourced populations, especially from the COVID-19 pandemic onward [73,75,76].

The potential for digital and mobile implementation of the tool represents a significant future direction. While this scoping review focused on synthesizing published research using the tool in its various applications, integrating such a validated tool into digital or mobile health platforms presents a considerable opportunity. Integrating this validated tool into digital or mobile health platforms could allow individuals to routinely input self-care behaviors, receive real-time feedback, and access personalized educational content, fostering greater engagement and adherence. For clinicians, such digital solutions could streamline assessment, enable remote monitoring, and facilitate timely interventions. Incorporating the HBP SCP into digital and mobile health interventions offers scalable and accessible avenues for improving cardiovascular health outcomes, warranting future research into its feasibility, usability, and effectiveness.

Several key correlates of HBP self-care emerged from the studies included in this review. Specifically, greater self-efficacy [27,28,56], stronger social support [35,56], and higher levels of health literacy [26,29] were associated with higher self-care behavior scores. As well, higher education was consistently associated with better self-care behaviors [30,31,36,39]. In contrast, comorbidities [30] and low income [33] were associated with lower self-care scores. Notably, Buco et al. [25] found that larger family size negatively affected disease self-management among community-dwelling older adults in the Philippines. A similar pattern was observed among US-residing Korean-American older adults with HBP [77]: those living alone—mostly residing in senior group housing—were at least twice as likely to have controlled BP compared to those living with others. Post-survey interviews revealed that frequent social interactions in senior group housing which involved encouragement about medication taking and health information sharing contributed to better HBP management [77]. With growing attention to the role of social determinants in cardiovascular outcomes [78], one of the studies included in the review [32] revealed that better neighborhood aesthetic quality and greater healthy food availability were associated with better self-care behaviors. Further, healthy food availability attenuated the negative impact of neighborhood violence on HBP self-care among socially disadvantaged Black or African American individuals. These findings point to the critical importance of both individual psychosocial and broader social factors in shaping HBP outcomes.

This review revealed the adaptation and application of the HBP SCP across diverse cultural and linguistic contexts. Thirteen published studies reported successful translation and validation of the tool in different languages, including Arabic [56], Persian [57], Turkish [59,68], Chinese [61], Indonesian [64,66], Korean [62], Vietnamese [67], and in Singaporean contexts [60,63,65], while demonstrating cultural relevance and strong evidence of validity and reliability. Notably, validity testing was conducted in various real-world healthcare settings such as community health centers, primary care, and outpatient clinics. Construct validity was robust and supported through exploratory and confirmatory factor analyses, although cultural relevance influenced factor loadings in some contexts—such as in Saudi Arabia, where smoking-related items on the HBP SCP were affected due to the cultural and religious unacceptability of smoking in Islamic tradition [56]. The results highlight the need for culturally informed implementation and interpretation when applying standardized tools like the HBP SCP across global populations.

Despite offering valuable insights into the global application and utility of the HBP SCP, this review has limitations. Cultural modifications, such as omitting items related to alcohol or smoking due to sociocultural norms, were often reported but rarely followed by full re-validation, raising potential concerns about construct equivalence across versions. To address these gaps, future research should prioritize standardized cross-cultural adaptation guidelines [79,80], while adopting established reporting guidelines. For instance, the recommendations by Streiner and Kottner [81] provide a comprehensive framework for reporting instrument development and testing, emphasizing clarity in methodology and results. Beyond linguistic translation, incorporating community-based participatory approaches can help integrate culture-specific contexts such as gender norms, literacy levels, and social support structures that may influence HBP self-care [80,82,83]. Future adaptations may also incorporate advanced psychometric methods like item response theory and Rasch modeling [84] to tailor HBP SCP to population-specific needs such as varying literacy levels while maintaining psychometric rigor. Another limitation is the possibility that some relevant studies may have been missed due to the limitations of the databases searched or variations in terminology used to describe the instrument. While a comprehensive search strategy was employed in consultation with a medical librarian, including multiple databases and manual searches, inconsistent naming conventions or indexing across studies may have limited the identification of all eligible publications.

## 5. Conclusions

The HBP SCP demonstrated its utility and cross-cultural adaptability, validity, and reliability, making it a suitable tool for evaluating HBP self-care behaviors, self-efficacy, and motivation across diverse populations and settings. Its demonstrated responsiveness in randomized trials affirms the value of HBP SCP as both a clinical and research tool. The successful cultural and linguistic adaptation of the HBP SCP in multiple regions further highlights its global applicability.

## Figures and Tables

**Table 1:** Characteristics of non-validation studies using HBP SCP and associated factors (*n* = 34).

First Author [Ref]	Design	Setting	Sample (Characteristic, *N*, Age)	Country/Language	Significant Factor Associated with HBP SCP
Acharya [22]	Cross-sectional study	Internal medicine and cardiac outpatient department in a hospital	Patients with HBP (*N* = 386) Mean (SD) age: 57.4 (11.3) years	Nepal/English	Behavioral score ~ religion, educational level, area of residence, income
AlHadlaq [23]	Cross-sectional study	Family medicine clinics	Patients with HBP (*N* = 187) 38% of patients were aged 51–70 years	Saudi Arabia/English	NR
Bahari [24]	Cross-sectional study	30 primary healthcare centers	Saudi men with HBP (*N* = 158) Mean (SD) age: 54.3 (15.0) years (range = 24–86)	Saudi Arabia/Arabic and English	↑ Self-efficacy score ~ ↑ behavioral score ↑ Family social support ~ ↑ self-efficacy score, ↑ behavioral score
Buco [25]	Cross-sectional study	Rural communities	Community-dwelling older adults (*N* = 304) Mean (SD) age: 69.4 (6.9) years	Philippines/Filipino	↑ Behavioral score ~ ↑ quality of life, ↓ family size
Boulware [48]	RCT	Academically affiliated community-based primary care clinic	African American patients with uncontrolled HBP (*N* = 159) Mean (SD) age: 57 (10.8) years	USA/English	Problem-solving intervention ~↑ behavioral score, ↑ self-efficacy
Darvishpour [26]	Cross-sectional study	Cardiac Care Unit (CCU) and post-CCU wards in public hospitals	Adults in Iran with HBP taking medication (*N* = 150) Age: NR	Iran/NR	↑ Behavioral score ~ ↑ older age, ↑ health literacy
Dorji [27]	Cross-sectional study	District hospital	Aged 18–60 years with HBP diagnosis for 6+ months, taking 1+ anti-hypertensive medication, and able to read and write in English with no history of stroke and psychiatric illness (*N* = 108) Mean (SD) age: 49.3 (8.4) years	Bhutan/English	↑ Self-efficacy score ~ ↑ lifestyle modifications
Ea [28]	Cross-sectional study	Community-dwelling Filipino immigrants	First generation Filipino immigrants in the US who have HBP (*N* = 162) 51% of the participant were 40–64 years	USA/English	↑ Self-efficacy score ~ ↑ hypertension self-care, ↑ patient activation
Gardiner [40]	Quasi-experimental study	University of Massachusetts Memorial Health Care system	Adults with a current diagnosis of HBP (*N* = 25) Mean (SD) age: 57 (12.1) years	USA/English	NR
Han [41]	Quasi-experimental study	Health fair and local market	Spanish-speaking Latinos in Baltimore, Maryland, who had uncontrolled HBP (*N* = 11) Median age (IQR): 54.7 (38.9–62.2)	USA/English	Health literacy-focused HBP intervention ~ ↑ self-efficacy score, ↑ behavioral score
Ho [29]	Cross-sectional study	Public primary care setting	Adults with HBP and chronic kidney disease in Singapore (*N* = 289) Mean [SD] age: 63.7 (8.7) years (range = 35–79)	Singapore/English	↑ Health literacy ~ ↑ self-efficacy score, ↑ behavioral score, ↑ motivation score ↓ Total score ~ male, ↓ health literacy
Jung [30]	Cross-sectional study	General hospital	Community-dwelling adults aged 65–91 years (*N* = 105) Mean (SD) age: 76.2 (6.5) years	South Korea/Korean	↑ Behavioral score ~ religious affiliation, ↑ education, marriage, living with family, ↑ cognitive function ↓ Behavioral score ~ ↑ comorbidities
Kim [31]	Cross-sectional study	Online survey representative of 49 states and DC	Adults in the US with diagnosis or symptoms of peripheral neuropathy (*N* = 608) Mean (SD) age: 63.1 (14.2) years	USA/English	Behaviors score ~ neuropathy pain total score
Lee [42]	Quasi-experimental study	Academic medical center	Patients with HBP receiving care at a cardiology clinic (*N* = 16) Mean age: 41 years (range = 26–55)	Korea/Korean	Discontinuing anti-hypertensive medication and providing education ~ ↑ behavioral score
Lunyera [32]	Cross-sectional study	Academically affiliated community-based primary care clinic in East Baltimore, Maryland	Black or African American individuals with social disadvantages with HBP (*N* = 159) Mean (SD) age: 57 (11) years	USA/English	↑ Behavioral score, ↑ self-efficacy score ~ ↑ perceived neighborhood health
Lynch [45]	Quasi-experimental study	Churches in a low-income, segregated neighborhood of Chicago	African Americans with uncontrolled HBP (*N* = 79) Mean (SD) age: 63.4 (12.5) years	USA/English	The church-based intervention trained community health workers to provide culturally tailored support ~ ↓ self-efficacy score
Moon [49]	RCT	Public primary care clinic serving the multi-ethnic population	Singaporean citizens or permanent residents aged between 40 and 70 years diagnosed with essential HBP (*N* = 928) Mean (SD) age: 56.3 (7.2) years	Singapore/NR	NR
Nazeri [33]	Cross-sectional study	Teaching hospital	Adult patients with HBP (*N* = 161) Mean (SD) age: 60.2 (17.0) years (range = 40–60)	Malaysia/Malay	Behavioral score ~ religion, race, monthly income, sources of information, and the existing place for exercise
Nursalam [34]	Cross-sectional study	NR	Adult patients with HBP with systolic BP > 160 mm Hg (*N*= 225) Age >25 years	Indonesia/NR	HBP self-care ~ coping skill
Pahria [35]	Cross-sectional study	Hospital clinics	Adult patients with HBP and complications (*N* = 102) Mean (SD) age: 56.4 (8.7) years	Indonesia/Indonesian	Behavioral score ~ medical history, family support, illness perception.
Rasdiyanah [43]	Quasi-experimental study	NR	Housewives with HBP (*N* = 66) Intervention: Mean (SD) age: 46.2 (8.2) years Control: Mean (SD) age: 46.3 (7.2) years	Indonesia/Indonesian	Face-to-face education and booklet ~ ↑ self-efficacy score
Rasyid [54]	Case-control study	Government hospital	Stroke and non-stroke patients (*N* = 24) Mean (SD) age: 56.4 (12.8) years	Indonesia/Indonesian	↓ self-efficacy score, ↓ behavioral score ~ ↑ risk of stroke
Rasyid [50]	Single-center, double-blinded, two-arm RCT	Government hospital	Patients with mild-to-moderate ischemic stroke, symptoms consistent with a vascular area of the brain, and a National Institutes of Health Stroke Scale score of 15 or lower (*N* = 120) Intervention: Mean (SD) age: 57.6 (13.3) years Control: Mean (SD) age: 57.3 (14.0) years	Indonesia/Indonesian	Stroke education before discharge with 2 refresher sessions ~ ↑ behavioral score, ↑ self-efficacy score
Salim [36]	Cross-sectional study	Primary care clinics	Adult patients with HBP representing multiethnic groups including Chinese, Malays, and Indians (*N* = 761) Mean (SD) age: 59.5 (10.2) years	Malaysia/English	↓ Behavioral score ~ men ↓ Total score ~ Chinese ethnicity ↑ Total score ~ ↑ education levels, positive family history of HBP
Talebi [51]	RCT	Comprehensive urban and rural health service centers	Adult patients with HBP with phone access (*N* = 82) Intervention: Mean (SD) age: 54.2 (9.6) years Control: Mean (SD) age: 54.2 (8.7) years	Iran/Persian	Telenursing intervention ~ ↑ behavioral score (categorized into four domains—diet, food labeling, drug regimen, and disease management)
Tan [37]	Cross-sectional study	NR	Malaysian adults without a formal HBP diagnosis (*N* = 260) Mean (SD) age: 32.0 (10.9) years	Malaysia/English, Malay (Bahasa), and Mandarin	Perceived severity, health motivation, perceived benefits, perceived barriers, self-efficacy, knowledge ~ behavioral score
Türkoğlu [44]	Quasi-experimental study	NR	Adult patients with HBP, at least a primary school education, and able to use a computer (*N* = 136) Web-Based: 45.7% in 30–40 years Face to Face: 33.3% in 30–40 years	Turkey/Turkish	Web-based and face-to-face patient education on the self-efficacy and health literacy ~ ↑ self-efficacy score
Visanuyothin [38]	Cross-sectional study	Urban community	Adult patients with an average clinic BP ≥ 140/90 mm Hg over the last three visits (*N* = 125) Mean age: 63 (range = 35–70) years	Thailand/Thai	NR
Visanuyothin [47]	Quasi-experimental trial	Urban community	Adult patients with HBP (*N* = 123) Intervention: Median age: 61.0 years (IQR: 8.0 years) Comparison: Median age: 63.5 years (IQR: 9.0 years)	Thailand/Thai	Community-based health literacy program ~ ↑ behavioral score
Wee [39]	Cross-sectional study	Urban community primary care clinics	Adults aged 40 and older with physician-diagnosed HBP (*N* = 1123; Malaysians = 702, Singaporeans = 421) Malaysians: Mean (SD) age: 60.4 (9.1) years Singaporeans: Mean (SD) age: 63.6 (9.7) years	Singapore and Malaysia/English, Malay and Mandarin	↑ Total score ~ Singapore ethnicity In Malaysia, ↑ behavioral score ~ aged 60 and above, Indian ethnicity, having tertiary education In Singapore, ↑ behavioral score ~ female, belonging to other ethnicities, having secondary education.
Yatim [46]	Quasi-experimental study	Three local non-governmental organizations	Adult patients with HBP and a body mass index of 18–32.5 kg/m^2^ (*N* = 45) Mean (SD) age: 60.1 (7.7) years	Malaysia/Malay	The structured, group-based hypertension self-management education program ~ ↑ behavioral score, ↑ self-efficacy score, ↑ motivation score
Zabler [52]	Two-group RCT	Free community clinic serving primarily low-income African Americans	African Americans on anti-hypertensive medication (*N* = 59) Intervention: Mean (SD) age: 53.9 (7.0) years Control: Mean (SD) age: 53.6 (6.4) years	USA/English	The Ecological Nurse Case Management intervention group ~ ↑ behavioral score
Zeng [53]	Pilot RCT	Village clinic in rural area	Adult patients with HBP, living with family, and speaking Mandarin or a local dialect (*N* = 44) Mean (SD) age: 63.1 (11.5) years	China/Chinese	The theory-based patient-family carer partnership intervention ~ ↑ behavioral score, ↑ self-efficacy score
Zeng [55]	Validation study	Village clinic in rural area	Adult patients with HBP living with family (*N* = 66 dyads of patients and family carers) Patients: Mean (SD) age: 66.8 (12.2) years Family carers: Mean (SD) age: 57.7 (11.5) years	China/Chinese	↑ Dyadic relationship score ~ ↓ self-efficacy score

HBP SCP, Hypertension Self-Care Profile; HBP, High Blood Pressure; BP, Blood Pressure; SD, Standard Deviation; NR, Not Reported; RCT, Randomized Controlled Trial; ↑ increased or higher, ↓ decreased or lower; ~ associated or correlated with.

**Table 2:** Characteristics of validation studies of HBP SCP (*n* = 14).

First Author [Ref]	Setting	Sample (Characteristic, *N*, Age)	Country/Languages	Scale	Translation	Psychometric Properties Test Used				
						Reliability Tests		Validity Tests		
						Test-Retest	Internal Consistency	Construct Validity	Content Validity	Criterion Validity
Bahari [56]	Primary healthcare centers	Saudi men aged 18 years or older with HBP (*N* = 160) 41.9% age 45–64 years old	Ahwaz, Iran/Arabic	behavior and self-efficacy	✓		✓	✓	✓	
Barati [57]	Community health centers	Older adults with HBP (*N* = 300) Age: NR	Ahwaz, Iran/ Persian	behavior and self-efficacy	✓		✓		✓	
Gheshlagh [58]	Public places in 22 regions of Tehran	Patients with HBP (*N* = 260) Mean (SD) age: 57.1 (16.1) years	Tehran, Iran/Persian	self-efficacy	✓	✓	✓	✓	✓	
Kes [59]	7 internal disease outpatient clinics of a university hospital	Patients with HBP (*N* = 200) Mean (SD) age: 58.3 (14.6) years	Turkey/Turkish	full scale	✓	✓	✓	✓	✓	
Koh [60]	Typical polyclinic (public primary care clinics) in Pasir Ris, an urban estate in the north-eastern region	Patients with HBP, aged 40+, and of Chinese, Malay, Indian, or other ethnic origin (*N* = 160 for test group, *n* = 71 for re-test group)	Singapore/NR	full scale		✓	✓			
Ma [61]	Tertiary hospital and community health center	Adults with HBP (*N* = 200)	China/Chinese	full scale	✓	✓	✓	✓	✓	
Na [62]	Senior welfare center	Older adults with HBP (*N* = 196) Mean (SD) age: 78.9 (6.4) years	South Korea/Korean	behavior	✓		✓	✓	✓	
Ngoh [63]	Primary care clinic	Adult patients with HBP (*N* = 153 for test group, *n* = 79 for re-test group) Median (IQR) age: 59 (52.0–65.5) years	Singapore/Chinese (Mandarin)	full scale	✓	✓	✓			
Salami [64]	Primary health care	Adult patients with HBP (*N* = 191) Mean (SD) age: 58.3 (10.2) years	Indonesia/Indonesian (Bahasa)	full scale	✓		✓	✓	✓	
Seow [65]	Public primary care clinic	Adult patients with HBP (*N* = 145) Age 41–70 years	Singapore/Malay	full scale	✓	✓	✓			
Upoyo [66]	Community	Patients with HBP (*N* = 40) Mean (SD) age: 61.7 (10.7) years	Indonesia/Indonesian	full scale	✓		✓		✓	
Van Troung [67]	Outpatient clinics of the Internal Medicine Department at a university hospital	Adult patients with HBP (*N* = 220) Mean (SD) age: 68.6 (13.4) years	Vietnam/Vietnamese	behavior	✓	✓	✓	✓	✓	
Versiyonunun [68]	Internal medicine outpatient clinic of a public hospital	Adult patients with HBP (*N* = 300) Mean (SD) age: 61.3 (11.0) years	Turkey/Turkish	full scale	✓	✓	✓	✓	✓	
Zhao [69]	Department of Cardiovascular Medicine of four tertiary referral centers	Older adult patients with HBP (*N* = 220) Mean (SD) age: 73.7 (9.0) years	China/Chinese	full scale	✓		✓	✓	✓	✓

HBP, High Blood Pressure; SD, Standard Deviation; NR, Not Reported; ✓ a specific psychometric test conducted or reported in the study.

## Data Availability

No new data were created or analyzed in this study. Data sharing is not applicable to this article.

## Supplementary Materials

The following supporting information can be downloaded at: https://www.mdpi.com/article/10.3390/ijerph22081244/s1, Table S1: Main findings of non-validation studies using HBP SCP (*n* = 34); Table S2: Psychometric properties result from validation studies of HBP SCP (*n* = 14).

## Abbreviations

The following abbreviations are used in this manuscript:

BP	Blood pressure
CI	Confidential interval
CFI	Comparative fit index
CVI	Content validity index
GFI	Goodness of fit index
HBP	High blood pressure
HBP SCP	Hypertension Self-Care Profile
ICC	Intraclass correlation coefficient
IFI	Incremental fit index
IQR	Interquartile range
KMO	Kaiser–Meyer–Olkin
NA	Not applicable
NR	Not reported
NHANES	National Health and Nutrition Examination Survey
NNFI	Non-normed fit index
OR	Odds ratio
RCT	Randomized controlled trial
RMSEA	Root mean square error of approximation
SD	Standard deviation
TLI	Tucker–Lewis index
US	United States
