# Supplementary material for: Use of the Hypertension Self-Care Profile: A Scoping Review
Source: Int J Environ Res Public Health. 2025 Aug 8;22(8):1244. doi: 10.3390/ijerph22081244 (PMC12386562; doi:10.3390/ijerph22081244)
Supplement: Supplementary file 1 [file ijerph-22-01244-s001.zip › ijerph-3683401-supplementary.pdf]

Supplementary data

Table S1. Main findings of non-validation studies using HBP SCP (n = 34).

1st Author [Ref]	Scale Used				Main Findings
	TOTAL	Behavior	Self-efficacy	Motivation	
Acharya [22]	NA	X	NA	NA	Poor self-care behavior found in 52.3% of participants. Significant associations with self-care behavior: Religion ($p < 0.001$), educational level ($p < 0.001$), area of residence ($p = 0.001$), and income ($p < 0.001$).
AlHadlaq [23]	NR	x (Cronbach's alpha = 0.90)	x (Cronbach's alpha = 0.90)	x (Cronbach's alpha = 0.91)	A low percentage of patients reported always or frequently engaging in HBP behaviors, such as physical activity (12.8%), reading nutrition labels (25.1%), replacing high-salt foods (35.8%), and checking blood pressure at home (37.9%). Higher rates were seen in non-smoking (65.2%) and regular doctor visits (85%). Patients were often motivated to limit high-salt condiments (56.7%), use healthier cooking methods (60.9%), practice non-smoking (67.4%), avoid stress (52.4%), and see their doctor regularly (85.0%). Self-efficacy was high for limiting salt (54.5%), healthy cooking (61%), non-smoking (65.2%), avoiding stress (52.4%), and doctor visits (85%).
Bahari [24]	NA	x (Cronbach's alpha = 0.84)	x (Cronbach's alpha = 0.90)	NA	Self-efficacy had a strong, positive correlation with self-care behaviors ($r = 0.68$, $p < 0.001$). Family social support showed moderate positive correlations with both self-care behaviors ($r = 0.35$, $p < 0.05$) and self-efficacy ($r = 0.38$, $p < 0.001$). In the multiple regression analysis, self-efficacy remained significantly associated with self-care behaviors ($\beta = 0.63$, $p < 0.05$)
Buco [25]	NA	x (Cronbach's alpha = 0.79)	NA	NA	HBP SCP behavioral scale positively influenced quality of life ($\beta = 0.15$, $p = 0.016$), while larger family size negatively affected behavioral scale ($\beta = –0.13$, $p = 0.034$).
Boulware [48]	X	X	X	X	The Problem-Solving intervention group had significantly higher odds of reporting both high self-care behaviors (OR = 18.7, 95% CI [4.0, 87.3]) and self-efficacy (OR = 4.7, 95% CI [1.5, 14.9]) at 12 months compared to baseline. They also had greater odds of achieving high self-care behaviors than the comparison group at 12 months (OR = 5.7, 95% CI [1.3, 25.5])
Darvishpour [26]	NA	X (Cronbach's alpha = 0.75)	NA	NA	Older adults had desirable self-care behaviors (mean [SD] = 51.8 [4.4]). Health literacy significantly predicted these behaviors ($\beta = 0.64$, $p = 0.000$), accounting for 40% of the variance ($\Delta R^2 = 0.404$).
Dorji [27]	NA	X	NA	NA	Participants occasionally practiced lifestyle modifications (mean score [SD]= 53.9 [7.7]), including physical activity, diet, weight reduction, alcohol moderation, stress management, and medication adherence (mean = 2.6–2.9). Smoking cessation had a slightly higher score (mean = 3.3). Knowledge ($\beta = 0.19$, $p = 0.04$) and self-efficacy ($\beta = 0.36$, $p < 0.001$) were key predictors, explaining 21.1% of the variance in lifestyle modifications.
Ea [28]	NA	NA	X	NA	HBP self-efficacy significantly predicted HBP self-care using the medical outcomes study specific adherence scale ($\beta = 0.27$, $p = 0.003$), contributing to 29.5% of the variance in self-care. HBP self-efficacy was positively correlated with self-care ($\beta = 0.78$, $p < 0.001$). Additionally, patient activation partially mediated this relationship ($\beta = 0.15$, CI 95% = 0.04, 0.32). The association between self-efficacy and patient activation was also significant ($\beta = 0.31$, $p < 0.001$).
Gardiner [40]	NA	NA	X	NA	In the Our Whole Lives group, which received an eHealth toolkit offering evidence-based lifestyle strategies, participants' self-efficacy increased from baseline (mean [SD] = 63.7 [9.1]) to follow-up (mean [SD] = 65.5 [7.6]); however, this change was not statistically significant ($p = 0.14$).

1st Author [Ref]	Scale Used				Main Findings
	TOTAL	Behavior	Self-efficacy	Motivation	
Han [41]	NA	X	X	NA	In the Health literacy-focused HBP intervention, HBP self- efficacy at baseline was 75 (IQR: 60–80), and at 16 weeks, it was 74 (IQR: 73–80), with a mean change of 3.8 (SD = 10.6) and an effect size of 0.4. HBP self-care behavior was 45 at baseline (IQR: 39–52) and increased to 67 at 16 weeks (IQR: 60–71), with a mean change of 17.3 (SD = 10.1) and an effect size of 1.7.
Ho [29]	X (Cronbach's alpha = 0.94)	X (Cronbach's alpha = 0.83)	X (Cronbach's alpha = 0.91)	X (Cronbach's alpha = 0.93)	Mean total self-care score was 182.7 (SD 23.2), with area specific scores as follows: the mean behavioral score was 55.3 (SD 8.6), the mean motivation score was 64.0 (SD 9.3), and the mean self-efficacy score was 63.3 (SD 8.7). Health literacy was significantly associated with all three self-care areas: behavior ($\beta = 0.30$, $P < 0.001$), motivation ($\beta = 0.40$, $p < 0.001$), and self-efficacy ($\beta = 0.38$, $p < 0.001$). Male gender and low health literacy scores were associated with lower total self-care scores.
Jung [30]	NA	X (Cronbach's alpha = 0.73)	NA	NA	Mean total behavior score was 57.8. Religious affiliation ($p = 0.020$), higher education ($p = 0.001$), marriage ($p = 0.007$), and living with family ($p = 0.002$) were associated with better self-care behaviors. Comorbidities negatively impacted health behavior ($p = 0.008$). Korean version of Montreal Cognitive Assessment scores (MoCA-K) were positively correlated with diet ($r = 0.27$, $p < 0.010$), health behavior ($r = 0.32$, $p < 0.010$), and total behavior ($r = 0.35$, $p < 0.001$).
Kim [31]	NR	X	NR	NR	HBP SCP behavioral score was significantly correlated with neuropathy pain total score ($p < 0.05$).
Lee [42]	NA	X	NA	NA	This study involved patients with well-controlled BP for at least six months on a single medication. After discontinuing their medication and providing education, they were monitored for six months with regular BP checks and self-care assessments. At the follow-up, self-care adherence increased in both normotensive and hypertensive groups over time ($p = 0.06$).
Lunyera [32]	NA	X	X	NA	The median (IQR) scores for HBP self-care behavior and self-efficacy were 50 (45-56) and 64 (57-72). Higher perceived neighborhood health was linked to better self-care behavior ($\beta = 2.48$) and self-efficacy ($\beta = 4.42$). Participants with better neighborhood health had higher self-care (51 vs 49) and self-efficacy scores (68 vs 61), and were more likely to score above 60 for both. The positive association between neighborhood aesthetic quality and HBP self-care behavior was more pronounced among participants with greater healthy food availability at home, showing a trend toward interaction ($p = 0.09$). The negative effect of neighborhood violence on self-care was reduced by higher healthy food availability ($p = 0.04$).
Lynch [45]	NA	NA	X (adapted, 10-item version with a 4-point Likert scale)	NA	The intervention is a church-based intervention trained community health workers (CHWs) from local African American congregations to provide culturally tailored support. The participants' HBP self-efficacy decreased significantly from 19.5 (SD = 5.3) at baseline to 11.8 (SD = 4.1) at follow-up, with a mean change of −7.9 (95% CI, −9.2 to −6.6). This decline in self-efficacy suggests that participants may have gained an understanding of what is needed to adopt a healthier diet, but through their attempts to make changes, they realized that maintaining a heart-healthy diet is more challenging than they originally expected.
Moon [49]	X	X	X	X	At baseline, the HBP SCP scores for participants in the logbook arm were as follows: mean (SD) score of 45.5 (9.1), 35.3 (9.2), and 37.9 (10.3) for behavior, motivation, and self-efficacy, respectively, and the cumulative score was 119.3 (SD = 25.6). In the smartphone app arm, the behavior score had a mean of 47.0 (SD = 7.79), motivation had a mean of 33.5 (SD = 9.11), self-efficacy had a mean of 38.1 (SD = 8.49), and the cumulative score was 118.7 (SD = 20.1). There were no significant differences between the two groups in any of the HBP SCP domains.

1st Author [Ref]	Scale Used				Main Findings
	TOTAL	Behavior	Self-efficacy	Motivation	
Nazeri [33]	NA	X	NA	NA	Mean (SD) self-care behavior score was 50.4 (8.2). Participants scoring below the mean were classified as having poor self-care, while those scoring 50.4 or above were considered to have good self-care. The characteristics such as religion (x^2:15.18; p <0.001), race (x^2:14.18; p <0.001), monthly income (x^2:6.77; p = 0.034), sources of information (x^2:9.87; p = 0.011), and the existing place for exercise (x^2:4.74; p = 0.030) significantly differed between those with poor and good self-care.
Nursalam [34]	NR	NR	NR	NR	HBP self-care was impacted by coping skill (p<0.001). Effective coping strategies enhanced self-care ability, improving HBP status (p=0.002).
Pahria [35]	X	X	X	X	Most patients with complicated HBP had high levels of self-care behaviors, with 55.9% showing good self-care behaviors and a mean score of 191.0 (SD = 5.2). Bivariate analysis found significant relationships between self-care behaviors and medical history (r = 0.47, p = 0.01), family support (r = 0.75, p < 0.01), and illness perception (r = 0.77, p < 0.01), but not knowledge (p = 0.19). Illness perception was the strongest predictor of self-care behaviors in multiple regression (β = 0.47, p <0.01).
Rasdiyanah [43]	NA	NA	X	NA	The intervention group received the face-to-face education and booklet. At baseline, the mean self-efficacy levels in both groups were similar (control = 54.55, intervention = 50.45). After the intervention, the self-efficacy in the intervention group increased to 59.91, while the control group saw a slight decrease to 54.15. The intervention group showed a mean increase of 9.46 in self-efficacy (p < 0.05). In contrast, the control group showed no significant change (-0.4, p > 0.05).
Rasyid [54]	NA	X (adapted, 17-item version with a 4-point Likert scale)	X (adapted, 17-item version with a 4-point Likert scale)	NA	This study used the Indonesian version of the HBP SCP, which adapted the HBP SCP by including both behavior and self-efficacy scales, with 17 items each. Stroke patients had significantly lower mean (SD) scores for behavior (22.4 [5.4]) and self-efficacy (21.7 [4.7]) compared to non-stroke patients (39.9 [6.1] and 45.0 [7.4], respectively; p < 0.001). Lower self-efficacy (OR: 0.75; p = 0.048) and self-care behavior scores (OR: 0.82; p = 0.018) were linked to a higher risk of stroke.
Rasyid [50]	NA	X (adapted, 17-item version with a 4-point Likert scale)	X (adapted, 17-item version with a 4-point Likert scale)	NA	This study used the same behavioral and self-efficacy scales, each consisting of 17 items, as described by Rasyid et al. [53]. The intervention group received stroke education before discharge, with refresher sessions at one- and three-months post-discharge. The control group received standard education. At baseline, there were no significant differences between groups in behavior or self-efficacy scores. At follow-up, the intervention group showed significant improvements in self-care behavior and self-efficacy. At one month, self-care increased by 4.56 points (95% CI: 0.57, 8.56) and self-efficacy by 4.95 points (95% CI: 0.84, 9.06). By three months, self-care further improved by 19.28 points (95% CI: 16.01, 22.56), and a 19.95-point increase in self-efficacy (95% CI: 16.61, 23.28).
Salim [36]	X	X	X	X	The mean total HBP SCP score was 124.19 out of 180 (68.9%). The lowest score was on the behavior scale, averaging 36.43 out of 60 (60.7%). Men had lower self-care scores than women. Chinese participants had lower HBP SCP scores than Malays and Indians. Higher education levels and a positive family history of HBP were associated with better self-care scores.
Talebi [51]	NR	X	NR	NR	This study used the HBP SCP to assess how telenursing impacts self-care behaviors. Self-care behaviors were categorized into four domains—diet, food labeling, drug regimen, and disease management. The intervention led to a 1.3-point increase in the mean score for a healthy diet (p=0.04) and a 1.2-point increase in disease management (p=0.004). However, the intervention did not significantly affect awareness of food labeling (p=0.38), or adherence to the medication regimen (p=0.62).

1st Author [Ref]	Scale Used				Main Findings
	TOTAL	Behavior	Self-efficacy	Motivation	
Tan [37]	NA	X (15 items only; 5 items irrelevant to the non-hypertensive group omitted	NA	NA	The average self-care behavior score was 39.3 (SD = 8.2). Reducing salt and calorie intake was the most challenging for 81.5% of participants. A multiple linear regression model with six predictors (perceived severity, health motivation, perceived benefits, perceived barriers, self-efficacy, and knowledge) explained 48% of the variation in self-care behavior ($R^2 = 0.49$, $F[6,231] = 38.54$, $p < 0.001$).
Türkoğlu [44]	NA	NA	X	NA	The intervention was web-based and face-to-face patient education on the self-efficacy and health literacy. The Self-Efficacy Scale pretest average (SD) score was 42.6 (18.1) for the web-based group and 37.3 (17.2) for the face-to-face group. Posttest scores increased to 57.9 (14.7) for the web-based group and 59.4 (12.3) for the face-to-face group. The study found that training improved patients' self-efficacy in managing HBP, regardless of the education method used.
Visanuyothin [38]	NA	X	NA	NA	Only 13% of HBP patients demonstrated adequate self-management behaviors, with one-quarter having high knowledge levels.
Visanuyothin [47]	NA	X 22-item behavioral scale	NA	NA	The community-based health literacy program intervention group showed a consistent increase in self-management behavior over the six-month period (mean = 2.4 at baseline and mean = 2.7 at 6-month, $p < 0.001$), while no changes were observed in the comparison group (mean = 2.4 at baseline and 6-month).
Wee [39]	X	X	X	X	Singapore participants had a significantly higher mean HBP SCP score (189.9, SD 27.6) compared to those in Malaysia (184.1, SD 22.8) ($p < 0.001$). Factors positively associated with HBP SCP behavior scores in both countries included being aged 60 and above, Indian ethnicity, and having tertiary education. In Malaysia, Malay ethnicity was linked to higher scores, while in Singapore, higher scores were also associated with being female, belonging to other ethnicities, and having secondary education.
Yatim [46]	X	X	X	X	The intervention was a structured, group-based hypertension self-management education (HSME) programme. The HBP SCP Scores showed significant improvements during the study. Behavior scores increased from 54.26 ± 8.79 at baseline to 59.89 ± 7.06 at 2 months ($p = 0.002$). Motivation rose from 64.93 ± 9.71 to 72.48 ± 5.53 at 2 months ($p = 0.001$), with a significant change at 1 week ($p = 0.005$). Self-efficacy improved from 63.59 ± 10.14 to 70.07 ± 7.13 at 2 months ($p = 0.002$), with an increase at 1 week ($p = 0.005$).
Zabler [52]	NA	X	NA	NA	The Ecological Nurse Case Management intervention group showed significant improvement in self-care behaviors at the 1-month follow-up ($\beta = 3.312$, $p = 0.001$), with continued gains at months 3 and 6 ($p < 0.001$). While the control group's scores rose initially at 1 month, they later declined. In contrast, the intervention group's scores increased steadily from 46.73 at baseline to 54.27 at 6 months.
Zeng [53]	NA	X	X	NA	The intervention was a theory-based patient-family carer partnership intervention. The HBP SCP scores for both behavior and self-efficacy showed significant improvements in the intervention group compared to the control group. Behavior scores increased from 45.2 (4.4) at baseline to 50.1 (4.9) post-intervention in the intervention group, with a significant interaction effect ($F = 17.668$, $p < 0.001$, $\eta p^2 = 0.457$). Self-efficacy improved from 51.1 (5.8) to 57.4 (6.3), also showing a significant interaction effect ($F = 5.996$, $p < 0.05$, $\eta p^2 = 0.222$).

1st Author [Ref]	Scale Used				Main Findings
	TOTAL	Behavior	Self-efficacy	Motivation	
Zeng [55]	NA	NA	X	NA	A significant negative correlation was found between dyadic relationship and HBP self-efficacy (Pearson's $r = -0.70$, $p < 0.001$).

HBP SCP, Hypertension Self-Care Profile; HBP, High Blood Pressure; BP, Blood Pressure; OR, Odds Ratio; 95% CI, 95% Confidence Interval; SD, Standard Deviation; IQR, Interquartile Range; NA, Not Applicable; NR, Not Reported; RCT, Randomized Controlled Trial. Note: The scale sections report Cronbach's alpha values, if available.

Table S2. Psychometric properties result from validation studies of HBP SCP (*n* = 14).

1st Author [Ref]	Country/ Languages	Scale	Translation Method	Psychometric Properties		Additional Results
				Reliability Tests	Validity Tests	
Bahari [56]	Ahwaz, Iran/ Arabic	behavior and self-efficacy	Forward-backward symmetrical translation by three nurses and one linguistics expert. Forward translation by 2 translators; backward by 2 different translators	**Internal consistency** tested by Cronbach α: self-care behavior (0.84) and self-efficacy (0.90)	**Content validity** obtained by experts. Cultural adaptations included removing the alcohol item and modifying references to pork and smoking, resulting in a revised score range of 19 to 76 for each scale.<br><br>**Construct validity**: Kaiser-Meyer-Olkin (KMO) of 0.83 and Bartlett test ($p \leq 0.001$). Factor loading on behavior scale (0.26–0.70) and self-efficacy (0.29–0.77).	Three items from the self-care behavior scale (Avoid smoking, continue taking blood pressure medication, and maintain a healthy weight) and one item from the self-efficacy scale (32: Avoid smoking) fell slightly below the acceptable threshold of 0.30.
Barati [57]	Ahwaz, Iran/ Persian	behavior and self-efficacy	Forward-backward translation: Forward translation by 2 translators; backward by 2 different translators	**Internal consistency** tested by Cronbach α: behavior (0.85) and self-efficacy (0.86).<br><br>**Test-retest reliability** tested by Intra-class Correlation Coefficient (ICC): behavior (0.89) and self-efficacy (0.92)	**Content validity** obtained by 15 experts including nutritionists, nursing assistants, senior staff assistants, and instrumentation assistants. The content validity ratio was greater than the cutoff value (≥49%).	A few items received lower content validity scores, particularly those related to alcohol consumption—likely due to cultural and religious sensitivities. In the self-efficacy scale, dietary items concerning saturated and solid fats were also reviewed, as older adults had difficulty understanding these terms. Additionally, some terms were adjusted, like using "antihypertensive drugs" instead of "blood-pressure drugs."
Gheshlagh [58]	Tehran, Iran/ Persian	self-efficacy	Forward-backward translation: Forward translation by 2 translators; backward by 2 different translators	**Internal consistency** tested by Cronbach α: self-efficacy (0.82)<br><br>**Test-retest reliability** tested by ICC of 0.93	**Content validity** obtained by 10 experts including nurses and psychologists, and researchers. Three items did not meet the required content validity index (CVI) score of 0.70 and were removed, including questions about reading food labels for salt content, choosing low-fat foods, and purchasing prescribed medications.<br><br>**Construct validity:** Exploratory factor analysis was carried out after deleting the 3 items. Factor loading on dietary (9 items, loadings 0.47–0.74), disease management (4 items, loadings 0.36–0.68), and adherence to treatment (4 items, loadings 0.56–0.63) were adequate. Confirmatory factor analysis showed good model fit ($X^2 = 328.35$, $p = 0.01$), with indices NNFI = 0.90, CFI = 0.91, IFI = 0.91, and RMSEA =	None

1st Author [Ref]	Country/ Languages	Scale	Translation Method	Psychometric Properties		Additional Results
				Reliability Tests	Validity Tests	
					0.082—all fall within acceptable thresholds, supporting the model's appropriateness.	
Kes [59]	Turkey/ Turkish	full scale	Forward-backward translation: Forward translation by 1 nurse professor translator; backward by 2 different translators (medical expert and bilingual language expert)	**Internal consistency** tested by Cronbach α: behavior (0.94), motivation (0.94), and self-efficacy (0.94)<br><br>**Test-retest reliability** tested by ICC: behavior (0.73), motivation (0.76), and self-efficacy (0.76)	**CVI** obtained by 12 nursing researchers with values of: Behavior (0.93), Motivation (0.91), and Self-efficacy (0.97)<br><br>**Construct validity**: The Behavior Scale explained 46.32% of the variance (factor loadings: 0.62–0.78), the Motivation Scale explained 45.87% of the variance (factor loadings: 0.54–0.80), and the Self-Efficacy Scale explained 48.08% of the variance (factor loadings: 0.57–0.77).	Item-total correlation: Behavior (0.54–0.74), Motivation (0.49–0.76), and Self-efficacy (0.53–0.73).<br><br>The Turkish HBP SCP was applied by the face-to-face interview method for the test–retest. In this study, 72% of the sample population were at an educational level of ≤ year 5 (primary level). It may be said that this Turkish version was reliable and valid for people with a year 6 or lower education level.
Koh [60]	Singapore/ NR	full scale	N/A (The English version was used)	**Internal consistency** tested by Cronbach α: behavior (0.86), motivation (0.95), self-efficacy (0.93), and total scale (0.96)<br><br>**Test-retest reliability** tested by ICC: behavior (0.67), motivation (0.76), and self-efficacy (0.72)	NR	Item–total correlations: Behavior (0.11–0.66), Motivation (0.40–0.81), Self-efficacy (0.35–0.79).<br><br>Results of the test–retest reliability evaluation of this HBP SCP tool suggest that it has potential application in English literate Asian patients with HBP as a majority of the Singapore population uses English as a common language.
Ma [61]	China/ Chinese	full scale	Cha's combined translation technique[77]: back-translation independently by 3 registered nurses, bilingual technique, group discussion, and pretest strategies	**Internal consistency** tested by Cronbach α: behavior (0.86), motivation (0.94), and self-efficacy (0.93).<br><br>**2-week Test-retest reliability** tested by weighted kappa statistic for each individual item: behavior (0.81–0.95), motivation (0.90–0.98), and self-efficacy (0.93)	**CVI** obtained by 7 experts with a value of 0.90 (Behavior), 0.95 (Motivation), 0.95 (Self-efficacy).<br><br>**Construct validity**: HBP SCP identified 6 factors in the Behavior Scale, with health promotion (63.15%) and habit modification (10.01%) as the primary contributors. The parallel result supported 1-factor solution for the Motivation Scale. The Self-Efficacy Scale identified 2 factors, with intake management contributing 44.30% and health maintenance which contributing 8.83%.	Significant differences between patients with or without comorbidities in behavior ($t = 2.55$, $p = 0.011$), motivation ($t = 3.25$, $p = 0.001$), and self-efficacy ($t = 3.02$, $p = 0.003$) supported the discriminative validity of Chinese HBP SCP.
Na [62]	South Korea/ Korean	behavior	Forward-backward translation by bilingual	**Internal consistency** tested by Cronbach α: Behavior scale (0.92)	**Construct validity** was tested by exploratory factor analysis. A two-factor solution was proposed and the two factors named, 'HBP-SC	None

1st Author [Ref]	Country/ Languages	Scale	Translation Method	Psychometric Properties		Additional Results
				Reliability Tests	Validity Tests	
			nursing professionals and a nutritionist		Diet behavior' and 'HBP-SC Health behavior (except diet)'. The two factors accounted for 48.9% of the variances.<br><br>**Concurrent validity** was tested by the correlation with HBP self-efficacy and the treatment compliance tool. The correlations were statistically significant with self-efficacy (r = 0.32, p < 0.001) and HBP treatment compliance (r = 0.39, p < 0.001).	
Ngoh [63]	Singapore/ Chinese (Mandarin)	full scale	Forward-backward translation by bilingual translators in Chinese and Malay	**Internal consistency** tested by Cronbach α: Behavior (0.84), Motivation (0.93), and Self-efficacy scale (0.93)<br><br>**Test-retest reliability** tested by ICC: Behavior (0.64), Motivation (0.58), Self-efficacy (0.71)	NR	Item–total score correlations: Behavior (0.06–0.68), Motivation (0.37–0.80), Self-efficacy (0.33–0.77).<br><br>The Mandarin HBP SCP demonstrated good internal consistency reliability, and test-retest stability among Mandarin-speaking patients. Its low ceiling effect suggests it effectively differentiates among diverse HBP patients.
Salami [64]	Indonesia/ Indonesian (Bahasa)	full scale	Forward-backward translation: Forward translation by 2 translators, a certified English translator and an English nursing lecturer at university; Backward translation by English translator	**Internal consistency** tested by Cronbach α: behavior (0.85), motivation (0.88), self-efficacy (0.86), and total scale (0.92)	**CVI** obtained by 5 experts (doctors, clinic nurses at primary health care, nursing lecturers, pharmacist, and anthropologists) with a value of 0.89.<br><br>**Construct validity**: KMO of 0.79, and Bartlett test of 1257.87 (p < 0.001). A single structure explained 55.85% of the total variance.	Item-total correlations: Behavior (0.237–0.649), Motivation (0.353–0.742), and Self-efficacy (0.302–0.642).<br><br>Mean (SD) of behavior = 57.5 (9.0), motivation = 66.0 (7.4), and self-efficacy = 60.7 (7.3). The study validated the results using WHO guidelines, with items showing excellent reliability and a satisfactory CVI score of 0.89. Experts recommended modifying three questions for clarity without altering content.
Seow [65]	Singapore/ Malay	full scale	Forward-backward translation by 2 independent professional and certified bi-linguists in English and Malay	**Internal consistency** tested by Cronbach α: behavior (0.85), motivation (0.93), and self-efficacy (0.95).<br><br>**Test-retest reliability** tested by ICC: behavior (0.66), motivation (0.66), and self-	NR	Mean (SD) of behavior = 66.4 (11.2), motivation = 65.8 (10.4), and self-efficacy = 50.3 (10.1).<br><br>Ceiling and Floor Effect: Self-efficacy had a ceiling effect of 15.2%. Other two subscales showed minimal ceiling effect (<15%). There was a minimal flooring effect but a

1st Author [Ref]	Country/ Languages	Scale	Translation Method	Psychometric Properties		Additional Results
				Reliability Tests	Validity Tests	
				efficacy (0.68). The response rate for the online test-retest was 43%.		borderline ceiling effect in the self-efficacy domain, limiting the tool's ability to distinguish high scorers. However, these individuals are not the primary focus for clinicians.
Upoyo [66]	Indonesia/ Indonesian	full scale	Forward-backward translation by 2 professional translators, both experts in the English language and health sciences	**Internal consistency** Cronbach α: behavior (0.96), motivation (0.92), and self-efficacy (0.91)	**CVI** obtained by 7 experts, including specialist doctor, nursing lecturer, health center nurse, nutritionists, public health expert, pharmacist, and sociologist, with a value of 0.96.	The Indonesian version of the HBP SCP questionnaire demonstrated excellent content validity. This version has strong content coverage and positive expert reviews, making it a valid and reliable tool for assessing behavior, motivation, and self-efficacy among HBP patients and for evaluating group-based HBP education programs in Indonesia.
Van Troung [67]	Vietnam/ Vietnamese	behavior	Forward-backward translation: Forward translation by 2 bilingual translators; Backward translation by a third bilingual translator	**Internal consistency** tested by Cronbach α: overall instrument (0.79), Subscales: advanced self-management skills (0.69), adverse health behaviors (0.82), medication adherence (0.76), diet-related knowledge regarding HTN (0.73), information skills (0.68). **Test-retest reliability**: The intraclass correlation coefficient between the scores of both applications was found to be r = 0.88.	**CVI** obtained by 5 Vietnamese nursing experts who had a PhD degree in nursing or public health and more than 5 years of experience in teaching nursing. I-CVIs of all items were from 0.85 - 1.00. The S-CVI/Ave was 0.95. **Construct validity** tested with confirmatory factor analysis. Fit indices—RMSEA = 0.07 (acceptable), CFI = 0.85, GFI = 0.88, TLI = 0.82 (slightly below good fit values).	The average variance extracted as follows: advanced self-management skills (0.35), adverse health behaviors (0.69), medication adherence (0.67), diet-related knowledge regarding HBP (0.31), and information skills (0.51). This study was limited by its focus on outpatient adults with HBP, potential influences from uncontrollable factors, and recall bias in responses such as reading of nutrition labels and eating less than 1 teaspoon of table salt per day. However, a sufficient sample size strengthened the factor analysis of the scale.
Versiyonun un [68]	Turkey/ Turkish	full scale	Forward-backward translation: Forward translation by 2 academics fluent in both Turkish and English; Backward translation by a third academic fluent in English	**Internal consistency tested** by Cronbach α: Behavior scale (0.81), Motivation scale (0.94), Self-efficacy scale (0.80). **Guttman split-half reliability coefficient**: Behavior scale (0.83),	**CVI** obtained by 8 experts (5 lecturers from the nursing department, 1 clinical nurse with a doctorate, 1 clinical nurse with a master's degree, and 1 specialist doctor). Mean CVI: Behavior scale (0.99), Motivation scale (0.98), Self-efficacy scale (0.96). **Construct validity** tested by confirmatory factor analysis with the following fit indices: RMSEA = 0.08; CFI = 0.94, GFI = 0.92, TLI = 0.94 for	Item–total score correlations: Behavior scale (0.37–0.58), Motivation scale (0.64–0.83), Self-efficacy scale (0.28–0.61). The variation in the Turkish HBP SCP structure compared to the original may stem from cultural, ethnic, racial, and social differences among the patients.

1st Author [Ref]	Country/ Languages	Scale	Translation Method	Psychometric Properties		Additional Results
				Reliability Tests	Validity Tests	
				Motivation scale (0.94), Self-efficacy scale (0.75).<br><br>**Test-retest reliability** tested by ICC: Behavior scale (0.99), Motivation scale (0.96), and Self-efficacy scale (0.97).	Behavior scale; RMSEA = 0.08; CFI = 0.92, GFI = 0.90, TLI = 0.90 for Motivation scale; and RMSEA = 0.07; CFI = 0.95, GFI = 0.92, TLI = 0.93 for Self-efficacy scale	
Zhao [69]	China/ Chinese	full scale	Used translated version from Ma et al., 2021 (Cha's combined translation technique[77]: back-translation independently by 3 registered nurses, bilingual technique, group discussion, and pretest strategies. Three bilingual registered nurses independently translate)	**Internal consistency** tested by Cronbach α: full scare (0.95), Behavior scale (0.93), Motivation scale (0.78), and Self-efficacy (0.917)<br><br>**Guttman split-half reliability coefficient**: 0.91	**Construct validity** of the HBP SCP scale was supported by principal factor analysis with Varimax rotation which extracted 7, 4, and 4 latent factors in three dimensions, explaining 66.05%, 64.87%, and 62.62% of the total variance, respectively.<br><br>**Criterion validity**: A strong correlation between the HBP SCP and Exercise of Self- Care Agency Scale found (r = 0.70). The Area Under the Curve for the HBP SCP was 0.84 (95% CI: 78.5–89.2), indicating good discriminative ability. The optimal cut-off point was 169.5, with a Youden's index of 54.89, yielding a sensitivity of 79.07% and specificity of 75.82%.	Item–total score correlations ranged 0.06–0.75 with the average correlation of 0.52. Item-to-total correlations mostly met recommended standards, except for a few items, which may be related to patients' differing concepts about each dimension. Both HBP SCP and Exercise of Self- Care Agency Scale are reliable, but HBP SCP is slightly more precise and effective for measuring self-care in older patients.

HBP SCP, Hypertension Self-Care Profile; ICC, Intraclass Correlation Coefficient; KMO, Kaiser-Meyer-Olkin; CVI, Content Validity Index; 95% CI, 95% Confidence Interval; SD, Standard Deviation; IQR, Interquartile Range; RMSEA, Root Mean Square Error of Approximation; CFI. Comparative Fit Index; GFI, Goodness-of-Fit Index; TLI, Tucker-Lewis Index.
